# Siewert II esophagogastric junction cancer: total gastrectomy or esophagectomy?

**DOI:** 10.1590/0102-67202025000019e1888

**Published:** 2025-08-04

**Authors:** Durval Renato WOHNRATH, Raphael de Oliveira e SILVA, Raphael Leonardo Cunha ARAUJO

**Affiliations:** 1 Barretos Cancer Center, Department of Upper Gastrointestinal and Hepato-Pancreatic-Biliary Surgery – Barretos (SP), Brazil.; 2 Hospital Ministro Costa Cavalcanti, Department of Surgery – Foz do Iguaçu (SP), Brazil.; 3 Universidade Federal de São Paulo, Digestive Surgery Unit, Department of Surgery – São Paulo (SP), Brazil.

**Keywords:** Stomach Neoplasms, Esophageal Neoplasms, Surgical Procedures, Operative, Surgical Oncology, Neoplasias Gástricas, Neoplasias Esofágicas, Procedimentos Cirúrgicos Operatórios, Oncologia cirúrgica

## Abstract

**Background:**

The surgical approach for esophagogastric junction cancers (EJC), Siewert II, has been controversial regarding margin control, reconstruction, and lymphadenectomy extension. Therefore, predicting the need for total/subtotal esophagectomy and proximal gastrectomy (TEPG) or total gastrectomy with distal esophagectomy (TGDE) can be challenging, with each direction usually excluding the other. Historically, complication rates for TEPG are higher, affecting further systemic treatment and long-term outcomes.

**Aims:**

The aim of this study was to describe a surgical strategy for approaching tumors such as Siewert II EGJ, with the intraoperative decision to perform total gastrectomy with lymphadenectomy D2 or esophagectomy with lymphadenectomy based on intraoperative frozen sections.

**Methods:**

All patients underwent laparotomy, beginning with greater curvature detachment while preserving the right gastroepiploic, right and left gastric arteries; dissection of the esophageal hiatus for node harvesting; and transection of the distal esophagus and its frozen section. TGDE was preferred if the proximal margin of the distal esophagus was negative; TEPG and gastric tube reconstruction were performed through transhiatal access if the margin was positive.

**Results:**

Among 38 Siewert II patients, 26 (69%) underwent TGDE and 12 (31%) underwent TEPG, regardless of the trend toward higher complication rates, positive margins, and shorter overall survival in the TEPG group, no statistically significant differences were detected.

**Conclusions:**

Although no significant differences in morbidity between the two procedures were noted, type II errors could be a possible cause. This study suggests that unnecessary esophagectomies can be avoided without jeopardizing surgical or oncologic outcomes by opting for a less morbid procedure.

## ARTICLE HIGHLIGHTS

- The surgical approach for esophagogastric junction cancers (EJC), Siewert II, has been controversial regarding margin control, reconstruction, and lymphadenectomy extension. Therefore, predicting the need for total/subtotal esophagectomy and proximal gastrectomy (TEPG) or total gastrectomy with distal esophagectomy (TGDE) can be challenging.
- Both surgical strategies are possible and have precise indications in each case. The TGDE may offer the best long-term quality-of-life perspective with less morbidity. This study suggests a stepwise strategy to approach lesions in EGJ, addressing lower morbidity since it prioritizes TGDE, grants a free margin, and does not jeopardize oncologic outcomes.
- Among 38 Siewert II patients, 26 (69%) underwent TGDE and 12 (31%) underwent TEPG, and regardless of the trend toward higher complication rates, positive margins, and shorter overall survival in the TEPG group, no statistically significant differences were detected.

## CENTRAL MESSAGE

The mainstream curative-intent treatment for resectable and nonmetastatic EGJ cancers is surgery, and often, in combination with chemotherapy, according to clinical and pathologic staging. In the case of Siewert II tumors, predicting the need for total/subtotal esophagectomy and proximal gastrectomy (TEPG) or total gastrectomy with distal esophagectomy (TGDE) can be challenging, with each direction usually excluding the other.

## PERSPECTIVES

Both surgical strategies are possible and have precise indications in each case. The TGDE may offer the best long-term quality-of-life perspective with less morbidity. This study suggests a stepwise strategy to approach lesions in EGJ, addressing lower morbidity since it prioritizes TGDE, grants a free margin, and does not jeopardize oncologic outcomes.

## VISUAL ABSTRACT

**Figure:**
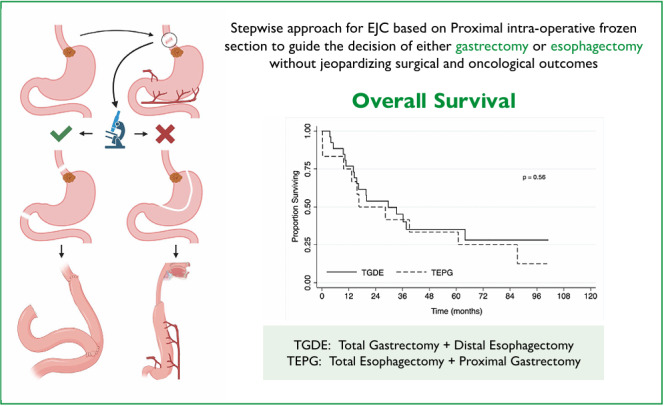

## RESUMO

**Racional:**

A abordagem cirúrgica para câncer da junção esofagogástrica (JEG), Siewert II, tem sido controversa em relação ao controle de margem de segurança, reconstrução e extensão da linfadenectomia. Portanto, prever a necessidade de esofagectomia e gastrectomia proximal (EGP) ou gastrectomia total com esofagectomia distal (GTED) pode ser um desafio. Historicamente, as taxas de complicações do EGP são mais elevadas, influenciando no tratamento adjuvante e os resultados em longo prazo.

**Objetivos:**

Descrever a estratégia cirúrgica para abordagem dos cânceres Siewert II da JEG, com decisão intraoperatória entre gastrectomia total ou esofagectomia, baseada em biópsias intraoperatórios.

**Métodos:**

Todos os pacientes foram submetidos à laparotomia, iniciando com descolamento da grande curvatura gástrica, preservando as artérias gastroepiplóica direita, gástrica direita e esquerda; dissecção do hiato esofágico e transecção do esôfago distal e biopsia de congelação. A GTED foi preferido se a margem proximal do esôfago distal fosse negativa e a EGP foi realizada por acesso transhiatal se a margem fosse positiva.

**Resultados:**

Entre 38 pacientes com Siewert II, 26 (69%) foram submetidos a GTED e 12 (31%) foram submetidos a EGP e, independentemente da tendência a maiores taxas de complicações, margens positivas e menor sobrevida global no grupo EGP, não houve diferenças significantes entre os grupos.

**Conclusões:**

A ausência de diferenças significativas na morbidade entre os dois procedimentos, tem o erro tipo II como possível causa. Este estudo sugere que esofagectomias desnecessárias podem ser evitadas sem comprometer os resultados cirúrgicos ou oncológicos e optando-se por um procedimento menos mórbido.

## INTRODUCTION

 Although gastric cancer represents the fifth most common cancer globally, with an estimated 952,000 new cases per year, and the third leading cause of cancer-related deaths, with approximately 726,000 (8.8%) deaths per year, the data on cancer affecting the esophagogastric junction (EGJ) are hardly evaluated separately^8^. 

 The mainstream curative-intent treatment for resectable and non-metastatic EGJ cancers is surgery, often in combination with chemotherapy, according to clinical and pathologic staging^1,12^. 

 The choice of an ideal surgical approach for lesions arising at the EGJ depends on the position of the tumor. According to Siewert’s classification, Type I is the adenocarcinoma of the lower esophagus (often associated with Barrett’s esophagus) with the epicenter located within 1–5 cm above the anatomic EGJ; Type II is the true carcinoma of the cardia at the EGJ, with the tumor epicenter within 1 cm above and 2 cm below the EGJ; and Type III is the subcardial carcinoma with the tumor epicenter within 2–5 cm below the EGJ, which infiltrates EGJ, and lower esophagus from below^20,21^. 

 According to the National Comprehensive Cancer Network, type II should be treated as an esophageal cancer using neoadjuvant chemoradiations^1,23^. 

 In the case of Siewert II tumors, predicting the need for total/subtotal esophagectomy and proximal gastrectomy (TEPG) or total gastrectomy with distal esophagectomy (TGDE) can be challenging, with each direction usually excluding the other. This study aimed to describe a possible surgical strategy for approaching Siewert II EJC, with the intraoperative decision to perform total gastrectomy with lymphadenectomy D2 or esophagectomy with mediastinal and retroperitoneal lymphadenectomy based on standardized intraoperative frozen section. 

## METHODS

### Patient and data collection

 The data, including demographic, clinical, operative, pathological, and follow-up information, were extracted from a prospectively maintained database of the Department of Upper Gastrointestinal and Hepato-Pancreato-Biliary Digestive Surgery at Barretos Cancer Hospital. This study was performed with the approval of the institutional board review according to internal policy for protected health information. All patients included in the study had EGJ adenocarcinoma preoperatively classified as type II tumors. The patients underwent TGDE or TEPG, performed by the same surgical team of the Barretos Cancer Hospital. 

 All patients have initially underwent laparotomy to determine the positive or negative esophageal margin, followed by transhiatal esophagectomy if negative margins were not achieved. The study included patients undergoing curative treatment based on physical examination, performance status, preoperative imaging including endoscopy, and computed tomography of the chest, abdomen, and pelvis. Patients were selected for D1 versus D2 lymphadenectomy based on age and performance status and according to surgeons’ judgment and the tumor board’s decisions. 

 Data on postoperative complications were retrieved retrospectively from medical records and classified according to the Dindo-Clavien classification for postoperative complications^6^. They were evaluated for the presence of any complication (grades 1–5 of Dindo-Clavien classification), the presence of major complication (grades 3–4), and death (grade 5) at 30 days. The estimated blood loss and transfusion rates were not fully available, and thus, they were not evaluated or described. 

### Operative technique

**Figure 1:**
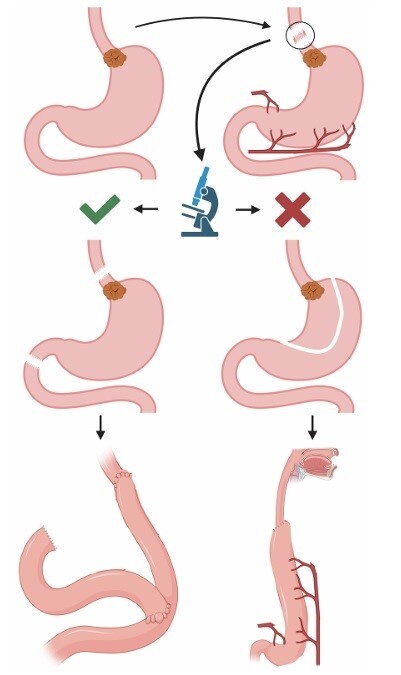
Standardization of stepwise surgical approach to esophagojejunal junction cancers: Step 1: exposure of distal esophagus tumor by abdominal access; Step 2: frozen section of distal esophagus above tumor location; Step 3: (two options) according to control of distal margin of the esophagus, if free margin is obtained distal esophaectomy and total gastrectomy is preferred, otherwise, total/subtotal esophagectomy and proximal gastrectomy is applied; Step 4: esophago-jejunal or esophagogastric anastomoses were done according to each type of resection, respectively, distal esophaectomy and total gastrectomy and total/subtotal esophagectomy and proximal gastrectomy.

**Figure 2:**
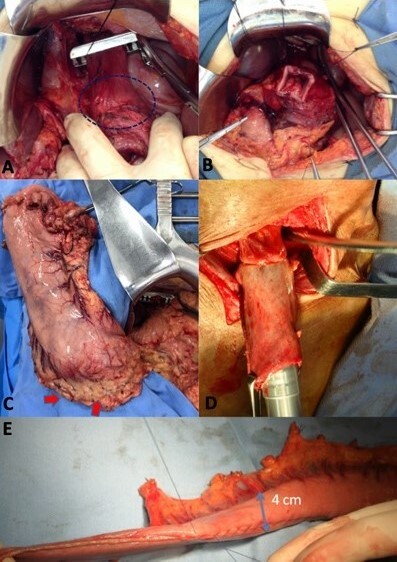
Intraoperative image sequence of the stepwise approach for Siewert II tumors; (A) exposure of distal esophagus with tumor (dashed ellipsis); (B) the esophagus margin preparation for frozen section; (C) stomach with preserved right gastroepiploic (red arrows), right and left gastric arterial arcades whereas the frozen section is made; (D) cervical esophagus-gastric anastomosis in case of positive margin in the distal esophageal frozen section, and (E) using a gastric tube nourished through right gastroepiploic and right gastric arterial arcades.

 All patients underwent surgery, beginning with greater curvature detachment while preserving the right gastroepiploic, right and left gastric arteries; dissection of the esophageal hiatus for node harvesting and transection of the distal esophagus and its frozen section were performed, as demonstrated in Figures 1 and 2. TGDE was preferred if the margin was free and the stomach vessels were finally ligated. However, if the margin was positive, TEPG was performed according to the international guidelines, and aimed lymphadenectomy was performed according to the technique preconized in our service, as previously published^26^. For total gastrectomy, D2 lymphadenectomy consisted of the removal of the superior omental bursa from the transverse mesocolon, greater and lesser omentum, perigastric nodal stations (1–6), and nodal stations of the left gastric artery (7), common hepatic artery (8a), celiac trunk (9), and proximal splenic artery (11p). The nodal stations of the splenic hilum (10) were only removed if a suspicious node was found and then removed en bloc with the spleen. Regarding the transhiatal esophagectomies, the lymphadenectomies were performed, including node stations 1, 2, 3, 4sa, 7, 8a, 9, 11p, and 110. 

### Statistical analyses

 Statistical analyses were performed using Fisher’s exact test and the Wilcoxon rank-sum test to examine categorical and continuous variables, respectively. Values are expressed as median (interquartile range) or percentage, as appropriate. The duration of follow-up for the overall survival (OS) analyses was the period, in months, between the date of the surgery and the date of the last follow-up recorded in the database or the date of death of the patient. A univariate analysis for OS probabilities was estimated using the Kaplan–Meier method and compared using the log-rank test. All analyses were performed using STATA software version 14.0 (Stata Corp, College Station, TX), and an alpha significance level of 5% (p<0.05) was used. 

## RESULTS

 A total of 38 patients with EJC were evaluated in our institution between 2009 and 2020. Notably, 26 (68%) patients underwent TGDE, and 12 (32%) underwent TEPG. Table 1 compares and summarizes the demographic and clinicopathologic characteristics of the two groups. Most of the patients were males with moderate- or high-grade tumors. 

**Table 1:** Demographic and clinicopathological distribution according to the type of procedure necessary to treat esophago gastric junction cancers (Siewert II).

	TGDE (%) n=26 (68)	TEPG (%) n=12 (32)	p-value
Median age*	60.5 (35–77)	55.5 (35–71)	0.12
Gender			
Male	20 (87)	9 (75)	1
Female	6 (13)	3 (25)	
ASA			
I	5 (19.2)	3 (25)	1
II/III	20 (77)	9 (75)	
N/A	1 (3.8)	.	
BMI	23.9 (16.8–37.4)	21.6 (16.5–29.9)	0.2
Histology			
G1	2 (7.7)	5 (41.6)	N/A
G2	11 (42.3)	3 (25)	
G3	11 (42.3)	2 (16.7)	
Signet ring	1 (3.7)	0	
Mucinous	2 (7.6)	0	
Other	.	2 (16.6)	

*Median (range)

†n=35.TGDE: total gastrectomy with distal esophagectomy;TEPG: total/subtotal esophagectomy and proximal gastrectomy;ASA: American Society of Anesthesiology score;BMI: body mass index.

**Figure 3:**
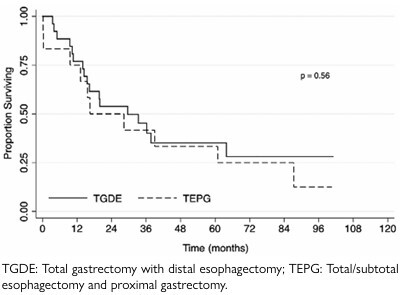
The Kaplan-Meier estimates of survival according to the type of surgery for esophagojejunal junction tumors.

 Concerning the types of surgery, Table 2 summarizes the surgical differences and outcomes of the two types of procedures. Approximately 92.3% of the patients who underwent TGDE had D2 lymphadenectomy, and all of them had an esophagojejunal anastomosis in a Roux limb configuration. All patients who underwent TEPG had a lymphadenectomy (preserving vasculature for the gastric conduit). The number of harvested nodes or positive nodes was not different. The majority of patients did not present any complications for TGDE (76.9%) or TGPE (58.3%), and no significant differences were detected among them. However, the presence of high-grade complications was 3.7 and 16.6%, respectively. Moreover, no differences in OS were detected, as described in Table 2 and depicted in Figure 3. The median follow-up for survival was 74.4 months. 

**Table 2:** Surgical outcomes according to the type of procedure necessary to treat esophagogastric junction cancers (Siewert II).

	TGDE (%) n=26 (68)	TEPG (%) n=12 (32)	p-value
Treatment			
Adjuvant	8 (30.8)	6 (50)	N/A
Preoperative	1 (3.8)	1 (8.3)	
Perioperative	6 (23.1)	0	
None	6 (23.1)	5 (41.7)	
Others	5 (19.2)	.	
Lymphadenectomy			
D1+	2 (7.7)	.	N/A
D2	24 (92.3)	.	
Regional lymphadenectomy (RL)	.	12 (100)	
Reconstruction			
Roux-en-Y	26 (100)	.	N/A
Gastric tube	.	12 (100)	
Number of harvested nodes^*^	28.2 (7–62)	25.6 (17–41)	0.56
Number of positive nodes^*^	4.9 (0–16)	9.2 (0–27)	0.16
Final free margin status	26 (100)	12 (100)	N/A
Presence of any complications			
Yes	6 (23.1)	5 (41.7)	0.27
No	20 (76.9)	7 (58.3)	
Presence of major complications (Dindo-Clavien grade 3–5)	1 (3.7)	2 (16.6)	0.23
Overall survival – months	44.2 (3.5–176.2)	37.9 (0.2–100.6)	
1-year – %	76.9	75	0.56
3-year – %	45.2	41.7	
5-year – %	35.2	33.3	

*Median (range).

TGDE: total gastrectomy with distal esophagectomy;TEPG: total/subtotal esophagectomy and proximal gastrectomy;N/A: non-applicable or non-available;RL: regional lymphadenectomy of node stations 1, 2, 3, 4sa, 7, 8a, 9, 11p, and 110.

## DISCUSSION

 The treatment of EGJ remains a challenge, and evolving changes have been made to the American Joint Committee on Cancer Eighth Edition^3,9^. Using this system, tumors from EGJ with an epicenter located at least 2 cm into the proximal stomach are now staged as gastric carcinomas. Tumors involving the EGJ with an epicenter=2 cm into the proximal stomach are still staged as esophageal carcinomas^9^. Considering the optimal curative-intent treatment for those patients, the FLOT4 trial, which uses perioperative triplet chemotherapy based on 5-fluorouracil, oxaliplatin, and docetaxel, is one of the cornerstones of the treatment of this disease^2,18^. Nevertheless, the efficacy of the treatment, with a median OS of 50 months for the FLOT group and 38 months for the 5-fluorouracil or capecitabine plus cisplatin and epirubicin group, shows the imperativeness of minimizing any complications postoperatively, permitting a fast return to chemoterapy. 

 Regarding surgical outcomes, treating all type II tumors as esophageal cancer with an esophagectomy may increase the comorbidity of the procedure, considering the wide range of complications (17–74%) reported in the literature^5,7^. The most concerns come from the fact that the TEPG usually includes a thoracic part, which induces more surgical trauma and, especially if an open esophagectomy is performed, is associated with an increased incedence of pulmonary complications^9,11^. 

 National audits and meta-analyses have demonstrated that in-hospital and 90-day mortality more accurately reflect actual mortality associated with esophagectomy, with in-hospital mortality being approximately 7–8% and 90-day mortality as high as 13% when assessed from all centers performing any annual volume of esophageal resections^15,24,25^. On the contrary, the expected complication rates for total gastrectomy in 30 day mortality vary from 4.7 to 5.4% in Western literature, approximately 1% in South Korea and Japan^4,13,16,19^. 

 Regarding the oncologic approaches and outcomes, in a recent systematic review, Heger et al. reported no difference in 5-year survival, 30-day mortality, and pathology results between esophagectomy and gastrectomy; however, there was an increased rate of morbidity after the TEPG compared with TGPE (39 vs. 57%, odds ratio: 1.55; 95% confidence interval 1.12–2.14; p=0.008; I2=0%)^10^. Moreover, another study has shown a better global quality of life, functional role (work and leisure), social function, and less fatigue after TGPE versus TEPG^14^. Even with minimally invasive techniques, which seem to attenuate some of these complications, the outcomes have been comparable to those of open esophagectomy, and the morbidity is not despicable^17,22^. 

 This study suggests a stepwise strategy to approach lesions in the EGJ, addressing lower morbidity with a free margin TGDE without jeopardizing oncologic outcomes. Nevertheless, it also represents such a small population, and perhaps it justifies the reason why no significant differences were detected in our sample. A possible type II error should be considered, especially when our results are compared to the current literature. Regarding surgical technique, this surgical strategy favors a stepwise approach to the cardia tumor, with stomach sparing and vascularization for an occasional gastric tube until free margins in the frozen section can be safely confirmed. Thus, in some cases with Siewert II tumors, unnecessary esophagectomies can be avoided without jeopardizing surgical or oncologic outcomes and opting for a procedure with less morbidity, according to the literature. 

 Our study is also related to other limitations, such as patient selection, which is usually common in retrospective and observational studies. The patient selection is perceptive, considering that a senior surgeon performed all the procedures; nonetheless, the decision of which patient should undergo this strategy was based on his judgment as well as on the departmental tumor board’s decision. Additionally, some patients underwent surgery without any neoadjuvant treatment/perioperative treatment, which is the gold standard today; nevertheless, it was not the standard addressing the period in which many patients were operated on upfront, especially for earlier stages of the disease. 

## CONCLUSIONS

 The best approach to standardizing curative-intent surgery for EGJ malignancies remains controversial, mostly due to the scarcity of evidence on this specific question. Perhaps, if both surgical strategies are deemed possible, a TGDE may offer the best long-term quality-of-life perspective with less morbidity. Thus, this study suggests a stepwise strategy to approach lesions in the EGJ, addressing lower morbidity since it prioritizes TGDE, grants a free margin, and does not jeopardize oncologic outcomes. 

## Data Availability

The informations regarding the investigation, methodology and data analysis of the article are archived under the responsibility of the authors.
